# Circadian and Homeostatic Modulation of Multi-Unit Activity in Midbrain Dopaminergic Structures

**DOI:** 10.1038/s41598-018-25770-5

**Published:** 2018-05-17

**Authors:** Karim Fifel, Johanna H. Meijer, Tom Deboer

**Affiliations:** 1 Department of Molecular Cell Biology, Neurophysiology unit, Leiden University Medical Center, P.O. Box 9600, 2300 RC Leiden, The Netherlands; 2 Present Address: International Institute for Integrative Sleep Medicine (WPI-IIIS), University of Tsukuba, 1-1-1 Tennodai, Tsukuba, Ibaraki 305–8575 Japan

## Abstract

Although the link between sleep disturbances and dopamine (DA)-related neurological and neuropsychiatric disorders is well established, the impact of sleep alterations on neuronal activity of midbrain DA-ergic structures is currently unknown. Here, using wildtype C57Bl mice, we investigated the circadian- and sleep-related modulation of electrical neuronal activity in midbrain ventral-tegmental-area (VTA) and substantia nigra (SN). We found no significant circadian modulation of activity in SN while VTA displayed a low amplitude but significant circadian modulation with increased firing rates during the active phase. Combining neural activity recordings with electroencephalogram (EEG) recordings revealed a strong vigilance state dependent modulation of neuronal activity with increased activity during wakefulness and rapid eye movement sleep relative to non-rapid eye movement sleep in both SN and VTA. Six-hours of sleep deprivation induced a significant depression of neuronal activity in both areas. Surprisingly, these alterations lasted for up to 48 hours and persisted even after the normalization of cortical EEG waves. Our results show that sleep and sleep disturbances significantly affect neuronal activity in midbrain DA structures. We propose that these changes in neuronal activity underlie the well-known relationship between sleep alterations and several disorders involving dysfunction of the DA circuitry such as addiction and depression.

## Introduction

Located in the mesencephalon, the ventral tegmental area (VTA) and Substantia nigra (SN) are the main sources of dopamine (DA) in the basal ganglia and forebrain^1^. Within these structures dopaminergic (70%), GABAergic (30%) and glutamatergic (2–3% only in the VTA) neurons are anatomically intermingled and electrophysiologically connected^2^. Functionally, these clusters of neurochemically diverse neurons control and/or modulate a broad range of behaviors including goal-directed behavior, motor actions, motivation, response to reward, learning, working memory, attention and decision-making^3^. Recently, growing interest has been shown towards the investigation of the role of VTA- and SN-dopaminergic and GABAergic neurons in the regulation of sleep and wakefulness^4–9^. This interest has been partially sparked by the recognition of sleep/wake cycle alterations in many neurological disorders in which VTA and SN function, including DA release, is compromised, such as Parkinson’s disease (PD)^10,11^. Inversely, alterations of the sleep-wake cycle are associated with risks for a wide variety of medical conditions directly or indirectly modulated by DA neurocircuitry^12–15^. Sleep deprivation (SD) adversely affects cognitive performances^16^, impairs judgment and decision making^16^, biases values computations by increasing the emphasis on gain outcomes relative to losses^17–21^, intensifies drug abuse and increases the likelihood of relapse after withdrawal^22,23^.

Recent animal studies as well as imaging studies in humans have shown that SD produces aberrant functioning in multiple sites of the dopaminergic reward circuitry and that these alterations were significantly correlated with SD-related behavioral and functional alterations^17–21^. Moreover, the modulation of the DA neurotransmission has been implicated in the therapeutic effects of SD in major depression^24^ as well as in the motor benefits experienced by a subset of patients with PD^25^.

The sensitivity of different brain structures to SD is variable^26–28^ suggesting that some brain structures belonging to the reward neuronal network might be more responsive to SD than others. Although some of the brain areas affected by SD are part of the DA circuitry^26–28^, the underlying mechanism(s) by which SD affects the mesocorticolimbic reward circuitry remain poorly understood. Additionally, whether sleep loss affects the electrophysiology of midbrain VTA and SN structures is unknown.

We therefore set out to assess the acute and long-term effects of a 6-hour sleep deprivation on electrical impulse frequency, also called multi-unit activity (MUA), as a measure of activity of neurons in the VTA and SN. In addition, electroencephalogram (EEG) and electromyogram (EMG) recordings were performed to investigate changes in the characteristics of sleep and waking before, during and after SD and their correlation with neuronal activity in VTA and SN. Given that SD affects DA neurotransmission^29^, we hypothesize that SD will alter the electrical activity of these midbrain structures.

## Materials and Methods

### Animals and locomotor activity recordings

The experiments were performed in rooms with monitored constant temperature and humidity conditions. Food and water were available ad libitum. A total of 17 adult male C57Bl/6JOlaHsd mice (16–20 weeks age old at the time of experiments) were used for this study. The animals were purchased from Harlan (The Netherlands). All the experiments were approved by of the Ethics Committee of Leiden University Medical Center and were carried out in accordance with the EU Directive 2010/63/EU on the protection of animals used for scientific purposes. Animal cages were equipped with passive infrared motion sensors to record general locomotor activity.

### *In vivo* multi-unit activity, EEG, and EMG recordings

*In vivo* multiunit activity (MUA) and EEG and EMG were recorded as described previously^30^. In brief, for the MUA recordings, stainless steel tripolar electrodes (0.125 mm diameter; Plastics One, Inc., Roanoke, VA) were implanted in each animal under deep anaesthesia. For differential recordings, two electrodes were directed toward the targeted midbrain structure with 0.4-mm space between the electrodes. The third electrode was placed in the cortex as a reference electrode. Measurements were performed from one electrode at a time. The electrodes were placed to record from the ventral tegmental area (VTA, relative to Bregma: 3.16 mm posterior and 0.59 mm lateral; depth: 4.37 mm), medial substantia nigra (SNM, relative to Bregma: 3.16 mm posterior and 1.11 mm lateral; depth: 4.22 mm) and lateral substantia nigra (SNL, relative to Bregma: 3.16 mm posterior and 1.75 mm lateral; depth: 3.7 mm). The coordinates were adapted from ref.^31^ (Supplemental Fig. S1).

For EEG, electrodes were screwed into the skull above the dura over the right cortex (2.0 mm lateral to the midline and 3.5 mm posterior to Bregma) and cerebellum (at the midline and 1.5 mm posterior to lambda). For EMG recordings, two wires with suture patches were inserted in the tissue between the skin and the neck muscle.

The animals were connected to the recording system via a flexible cable and counterbalanced swivel system, and the animals were acclimated to the setup under similar (12:12 L:D, food ad libitum) conditions. The animals’ behavioural activity (drinking and locomotion) was recorded continuously in order to obtain an estimate of the circadian rhythm.

Neuronal activity in the midbrain structures was amplified approximately 40,000X, band-pass filtered (500–5,000 Hz, −40 dB/decade). Online, a window discriminator converted the action potentials into electronic pulses. A second window discriminator was set at a higher level to detect artefacts caused by the animal’s movements. Action potentials and movement-related artefacts were counted in 10-s epochs. The analogue EEG and EMG signals, which were recorded continuously, were amplified approximately 2,000X, band-pass filtered (0.5–30 Hz, −40 dB/decade), and digitized at 100 Hz. All data were recorded simultaneously and stored on a computer hard disk. The stability of the multi-unit signal and EEG recording was evaluated daily by visually inspecting the signals. The circadian rhythm in the signal and the amplitude of the EEG were monitored before the baseline data were collected. As soon as the signals were stable, experimental recordings were started. After the experiments, the animals were sacrificed to verify the recording sites. To mark the location of the electrode tip, current was passed through the electrode, and the brain was perfused with a buffered solution containing 4% paraformaldehyde and 8% potassium ferrocyanide.

The brains were removed, post-fixed overnight in 4% paraformaldehyde, and cryo-protected in 30% sucrose solution. Free-floating coronal sections (40 μm thickness) were cut on a freezing microtome. The sections were stained with cresyl violet, mounted on gelatinized slides, dried, dehydrated in increasing gradients of ethanol, cleared in toluene, and cover-slipped with Depex.

Offline, the EEG power density spectra were calculated in 10-s epochs corresponding to the 10-s epochs of the action potentials of the targeted hypothalamic structure using a fast Fourier transform (FFT) routine within the frequency range of 0–25.0 Hz in 0.1-Hz bins. EMG signals were integrated over 10-s epochs. Three vigilance states—wakefulness, NREM sleep, and REM sleep—were determined visually based on standardized EEG/EMG criteria for rodents^30^. Wakefulness was scored when the EMG showed an irregular, high-amplitude pattern and the EEG signal was low in amplitude with relatively high activity in the theta band (6–9 Hz). NREM sleep was scored when EMG amplitude was low, and the EEG amplitude was higher than during wakefulness, with high values in the slow wave range (1–4 Hz). REM sleep was scored when the amplitude of the EMG and EEG were low, and the EEG showed relatively high values in the theta range. Epochs containing artefacts in the electrical signal or in the EEG signal (observed during the scoring of the vigilance states) were excluded from the analysis of the neuronal activity and EEG spectral analysis. Over the 3 days of recordings, on average 31.4 (±9.7) 10-seconds epochs per day and per animal were excluded because of electrical artefacts of the signals. This correspond to 2.2% of the entire signal analysed per animal.

All MUA data and EEG power density data were calculated relative to the respective mean values recorded during NREM sleep over the 24-h baseline L:D period. This enabled us to calculate mean values over all animals. To analyse changes of EEG power density in SWA and changes in neuronal activity at vigilance state transitions, intervals with a duration of 4min containing artefacts-free transitions from one vigilance state (VS1) to another (VS2) were selected by the following criteria^30^: (A) In the 2 mins preceding the transition, at least 75% had to be scored as VS1, and not more than two epochs of VS2 should occur. (B) In the 2 mins after the transition, at least 75% had to be scored as VS2. (C) Furthermore, the three 10-s epochs preceding and following the transition had to belong to the vigilance state corresponding to the transition.

### Sleep deprivation

A previously validated method using an enriched, novel environment^32^ was used to stimulate spontaneous exploratory wakefulness without inducing stress. SD was performed during the first 6 hours of the light-dark cycle. The duration of 6 h was chosen to avoid potential stress effects that might be precipitated following a longer period of SD. Clean bedding, food, water, toys, and novel nesting materials were used as stimuli to stimulate wakefulness. During the SD episode, the animals were monitored via their online EEG signal. Whenever the animals appeared to be entering NREM sleep—or if an increase in slow wave amplitude was observed—new material was introduced to the cage of the animal.

### Statistical analysis

**Figure 1:**
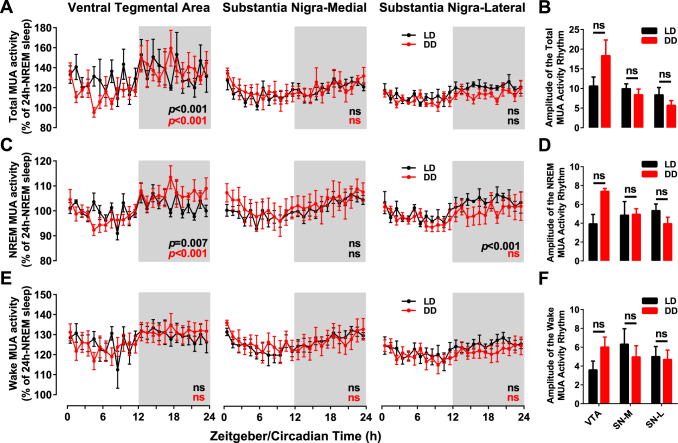
Daily and circadian modulation of MUA means in the VTA, SNM and SNL over respectively Light-Dark (LD, Black) and constant darkness (DD, red) cycles. Total (**A**), NREM sleep related (**C**) and wakefulness-related (**E**) MUA are displayed in 1-hour bins as a percentage of the mean activity during NREM sleep over LD cycle. The light and dark phases of LD cycles are indicated by the white and grey backgrounds respectively. (**B**,**D**,**F**) show the cosinor amplitudes of MUA rhythms. Error bars represent s.e.m. ns: not significant. (One-way ANOVA and Cosinor analysis in **A**,**C**,**E** and t-test in **B**,**D**,**F**).

**Figure 2:**
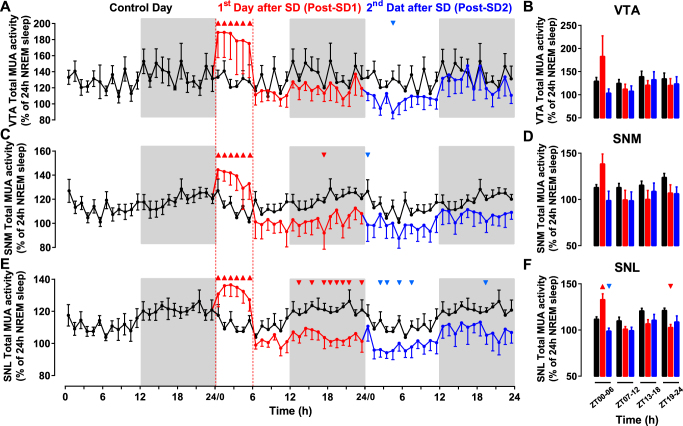
Long-term effects of 6 hours of sleep deprivation (SD) on neuronal activity in the VTA and SN. (**A**,**C**,**E**) Time course of mean neuronal activity in the ventral tegmental area (VTA, n = 6), medial substantia nigra (SNM, n = 5) and lateral substantia nigra (SNL, n = 6) measured over three consecutive 24-hour periods. Activity is displayed in 1-hour intervals as a percentage of the mean activity measured during NREM sleep during the baseline day. SD was induced during the first 6 hours of the first day after baseline, and activity was measured during the 1^st^ (red) and 2^nd^ (blue) days after SD. For comparison, the data recorded during the baseline day in (**A**,**C**,**E**) are triple-plotted (black lines). The light and dark phases of LD cycles are indicated by the white and grey backgrounds respectively. During SD and in both the VTA (**A**) and SN (**C**,**E**) neuronal activity increased significantly compared to baseline. Triangles indicate significance with *p* < 0.05 (two-way repeated measures ANOVA followed by Dunnett’s test). (**B**,**D**,**F**) Relative mean neuronal activity measured in 6 h-bins in the VTA (**B**), SNM (**D**) and SNL (**F**) during control day (black bars), Post-SD1 (red bars) and Post-SD2 (blue bars). VTA, Ventral Tegmental Area; SNM, Medial Substantia Nigra; SNL, Lateral Substantia Nigra.

**Figure 3:**
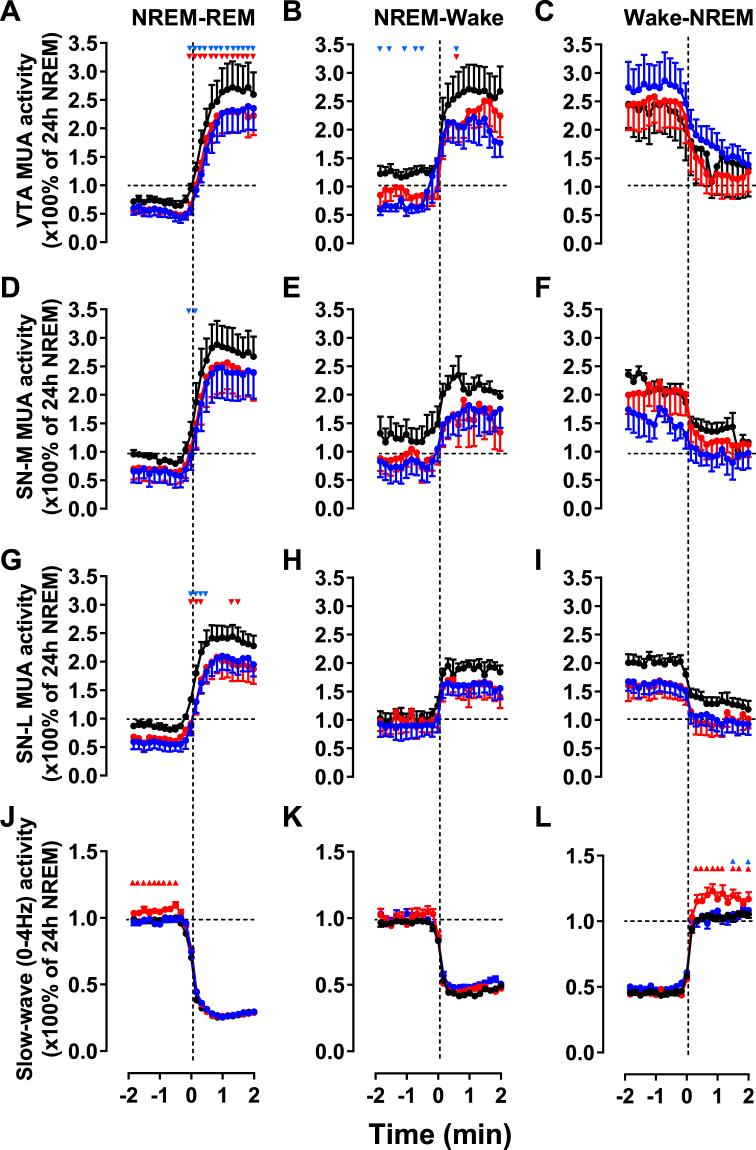
Multi-unit activity during vigilance state transitions during baseline (black), SD day (red) and post-SD (blue). Time course of the VTA (**A**–**C**), SNM (**D**–**F**) and SNL (**G**–**I**) neuronal activity and EEG slow-wave activity (power density 0–4 Hz) at the transition from NREM to REM sleep (**A**,**D**,**G**,**J**), NREM to REM sleep (**B**,**E**,**H**,**K**) and wake to NREM sleep (**C**,**F**,**I**,**L**) during the 2 min before and after the vigilance state transition. The curves connect 10s mean-values calculated over the entire LD, SD and post-SD days. All variables are expressed as a percentage of the mean activity during NREM sleep over baseline. All changes at the transition were significant (*p* < 0.001, ANOVA factor ‘time’ over 24 10-s epochs). Black lines: Control day; Red lines: Post-SD1; Blue lines: Post-SD2. Additionally, red and blue triangles indicate significant changes of neuronal activity during SD and post-SD days (two-way repeated measures ANOVA followed by Dunnett’s test).

**Figure 4:**
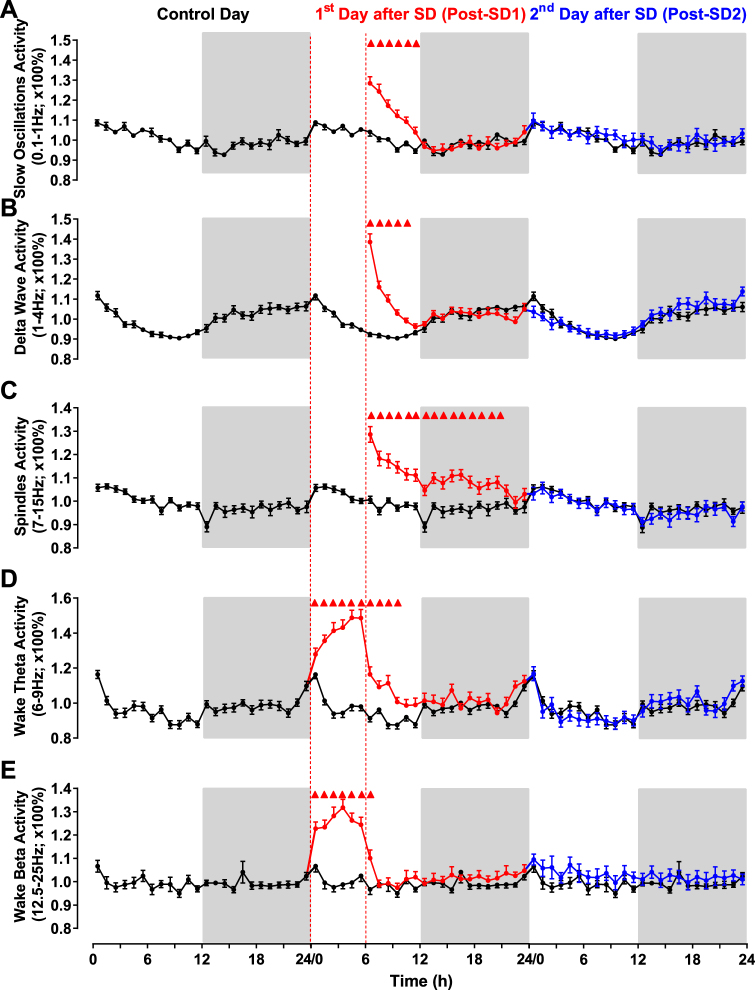
Changes in cortical EEG power density following 6 h SD. (**A**–**E**) Time course of mean percentages of EEG slow oscillations activity (**A**), Delta wave activity (**B**), spindle frequency activity (**C**) during NREM sleep, and theta activity (**D**) and beta activity (**E**) during waking over the 72 h experimental protocol. Data are presented as a percentage of the mean activity (±s.e.m.) over the 24 h baseline day and averaged in 1h bins. The traces of the baseline day are triple-plotted in black for easy comparison. Values during the 6 h SD in (**A–C**) are omitted because the remaining ≈10% of NREM during this period are influenced by the SD intervention. The light and dark phases of LD cycles are indicated respectively by white and black shading in the background. Triangles indicate significance with *p* < 0.05 (two-way repeated measures ANOVA followed by Dunnett’s test).

**Figure 5:**
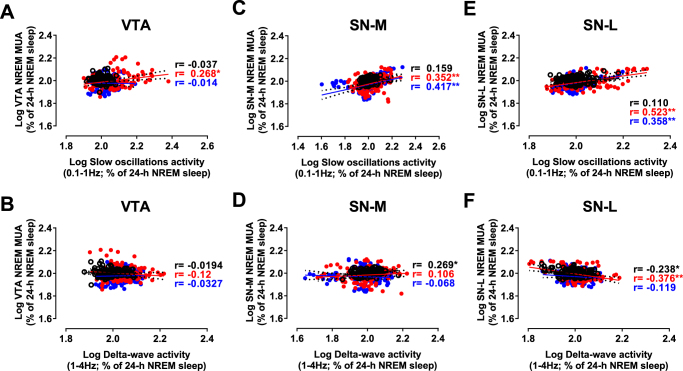
Correlations between neuronal activity in the VTA (**A**,**B**), SNM (**C**,**D**) and SNL (**E**,**F**) and slow oscillations and delta wave activities measured from EEG signals during NREM sleep. Changes in mean firing rates in the VTA, SNM and SNL measured in 1-min bins as a function of the power density of slow oscillations (**A**,**C**,**E**) and delta waves (**B**,**D**,**F**). *p < 0.05, **p < 0.01, (Black circles: Control day; Red circles: Post-SD1; Blue circles: Post-SD2).

**Figure 6:**
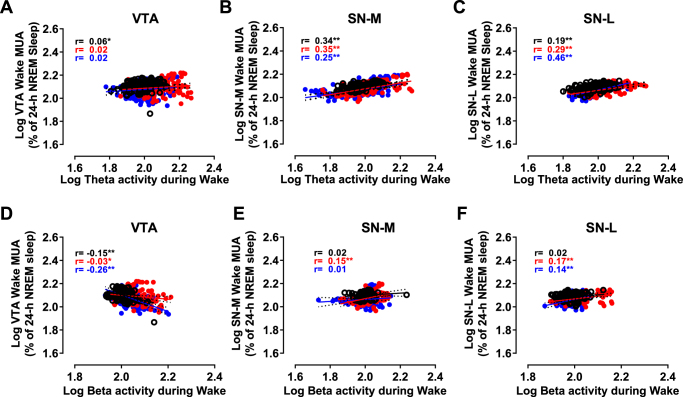
Correlations between neuronal activity in the VTA (**A**,**B**), SNM (**C**,**D**) and SNL (**E**,**F**) in one hand and theta (**A**–**C**) and beta (**D**–**F**) activities measured from EEG signals during wakefulness. (**A**–**F**) Changes in mean firing rates in the VTA (**A**,**D**), SNM (**B**,**E**) and SNL (**C**,**F**) measured in 1-min bins as a function of the power density of theta activity (**A**–**C**) and beta activity (**D**–**F**). *p < 0.05, **p < 0.01 (Black circles: Control day; Red circles: Post-SD1; Blue circles: Post-SD2).

Data were analysed using SigmaStat version 12.0. All summary data are reported as the mean ± s.e.m. Statistical significance was determined using one-way ANOVA (Fig. 1A,C,E; Fig. S2A–H; Table 1; Fig. S5A–L), paired Student’s *t*-test (Fig. 1B,D,F), cosinor analysis (Fig. 1A,C,E, Fig. S2A–H), a repeated-measures ANOVA, with time, neuronal activity, sleep state, and power density considered as repeated measures coupled to Dunnett *post hoc* analysis in the case of significance (Fig. 2A–F; Fig. 3A–L; Fig. 4A–E; Fig. S6A–F; Fig. S7A–F; Fig. S8A–F; Fig. S9A–F), second order polynomial regression (Fig. 4; Fig. S2) or simple linear regression (Fig. S4A–F; Fig. 5A–F; Fig. 6A–F). *p*-values are indicated in the text and the figure legends. Differences were considered significant when *p* < 0.05.

## Results

### Daily and circadian modulation of MUA in the VTA and SN

Baseline recording of both EEG/EMG polysomnography and MUA in VTA and SN were obtained under both 1 day of light/dark (LD) cycle followed by 1 day of constant darkness (DD). As previously shown^33^, mice spent 62.3% of their time asleep during the light- and subjective day-periods in LD and DD respectively (Supplemental Fig. S2A–C). Cortical EEG power densities (between 0–25 Hz) also showed a dynamical change over time in LD and DD (Supplemental Fig. S2D–H). The EEG slow-wave activity (SWA, EEG power between 0.1 and 4 Hz during NREM sleep) is considered to be the best physiological indicator of sleep pressure^33–35^. It peaked in early rest phase and decreased progressively to reach low levels in late rest phase at the transition to the active phase in both LD and DD. During the active phase, sleep pressure builds up^34^ as evidenced by the progressive increase in SWA. In both LD and DD, it reached a maximum at the transition to the rest phase (LD: R^2^ = 0.7, p < 0.0001; DD: R^2^ = 0.64, p < 0.0001, second order polynomial regression analysis, Supplemental Fig. S2D, E). Recently, EEG power in the theta and higher beta (15–35 Hz) were shown to track sleep need during wakefulness^36,37^. We therefore extracted power density from waves in this frequency range from the EEG signals during wakefulness and analyzed their evolution over LD and DD. We found that the dynamic of the power density of theta activity (6–9 Hz) during waking in both LD and DD parallels the evolution of SWA in NREM sleep (Supplemental Fig. S2G) consistent with published data in rats^36^. In addition, power density in the low beta range (12.5–25 Hz) showed no significant circadian modulation (Cosinor analysis, Supplemental Fig. S2H) although two-way ANOVA revealed significant differences over the day between LD and DD (*p* = 0.002, Supplemental Fig. S2H).

**Table 1:** Vigilance states modulation of neuronal activity in the VTA, SNM and SNL.

Midbrain Structure	Wakefulness	REM Sleep
SN-Lateral (n = 6)	137.4 ± 4.1*	141 ± 4.6*
SN-Medial (n = 5)	145.2 ± 6*	149.1 ± 9.1*
VTA (n = 6)	183.7 ± 28.7*	145.5 ± 6.2*

MUA activity is calculated as a percentage of the mean activity during NREM sleep over 24 h (set as 100). All midbrain structures increased their neuronal firing rate during wakefulness and REM sleep. *p < 0.001.

MUA recording were performed in the VTA (n = 6) and SN (n = 11). DA neurons within the VTA and SN are molecularly, anatomically and functionally heterogeneous^38^. Two populations of DA neurons, corresponding to medial-SN and lateral-SN, have been recently identified based on molecular as well as electrophysiological characteristics^39,40^. We therefore, distinguished between medial and lateral recordings obtained from SN (SNM, n = 5 and SNL, n = 6 respectively). In the VTA, a significant daily (in LD) and circadian (in DD) modulation of MUA was found with high levels during the dark phase and subjective night and low levels during the light phase and subjective day (*p* < 0.001, One-way ANOVA and cosinor analysis, Fig. 1A) in phase with the behavioral rhythm of the animals (Fig. 1A, Supplemental Fig. S2A). No circadian modulation of MUA was found under either of the conditions in the SN region (One-way ANOVA and cosinor analysis, Fig. 1A). Examples of individual raw data of MUA are shown in Supplemental Fig. S3.

A vigilance state-dependent modulation of neuronal activity in both VTA and SN was found (Table 1, Supplemental Figs S4, S5). The firing rates were higher during wakefulness and REM sleep compared to NREM sleep (Table 1, Supplemental Fig. S4). This differentiation of firing rates between states, together with the nocturnal distribution of sleep and waking could be the cause of the circadian modulation of MUA. On the other hand, intrinsic changes in neuronal activity could also result in a circadian modulation of MUA. To disentangle these two factors, we analyzed the patterns of neuronal activity separately for waking (Fig. 1C) and NREM sleep (Fig. 1E). The VTA was the only structure that showed a daily and circadian modulation of total MUA (*p* < 0.001, One-way ANOVA, Fig. 1A). During NREM sleep, VTA and SNL showed a significant modulation during NREM sleep in both LD and DD while the modulation in SNM was not significant (Fig. 1C). None of the structures showed a significant daily or circadian modulation during waking (Fig. 1E). To compare the rhythms between VTA and SN, we extracted the amplitudes from the Cosinor fits of each individual rhythm (Fig. 1B,D,F). Unlike the robust rhythms of MUA in the SCN^41^, the amplitude of MUA rhythms we recorded in the VTA and SN were lower (Fig. 1B,D,F) and were comparable to the published MUA recordings in the SN in rats^42^. We found no difference in the robustness of the MUA rhythms between LD and DD under all vigilance-state conditions (Fig. 1B,D,F).

### SD induces long-lasting alterations of MUA in the VTA and SN

To assess the consequence of increased sleep pressure on the MUA of VTA and SN, mice were challenged with a 6 h sleep deprivation. As shown before^33,34^, the animals responded to the SD by spending 6.4% more time in sleep (NREM and REM sleep) during the 16 h following SD. In the 2^nd^ day following SD, the animals fully recovered as values returned to baseline levels (Supplemental Figs S6 and S7).

During SD, all investigated midbrain areas showed an increase in neuronal activity (Fig. 2A,B,C), and after SD, a sustained decrease in activity was observed. In both SN areas, neuronal activity dropped after SD to levels below control. Although this decrease did not reach significance in all time points, this decrease in activity lasted during the whole 42 h recorded following SD and pre-SD levels were not fully recovered mainly in SNL (Fig. 2B,C). No significant alterations of MUA rhythms were found on the 2^nd^ day following SD (Cosinor analysis, P > 0.05, Fig. 2A,B,C).

Because changes in vigilance states influenced neuronal firing rate (Table 1, Supplemental Figs S4, S5) and SD induced alterations in sleep/wake architecture (Supplemental Fig. S6), the alterations observed in the MUA of the VTA and SN (Fig. 2) could be due to either changes in sleep/wake distribution or intrinsic changes in electrical activity caused by SD, or both. To discriminate between these two factors, we analyzed the patterns of neuronal activity separately for waking and NREM sleep (Supplemental Figs S8 and S9). We found that the decrease in MUA in the VTA and SN after SD was evident both during wakefulness (Supplemental Fig. S8) and NREM sleep (Supplemental Fig. S9). Notably, the decrease in MUA was maintained even after the normalization of the vigilance states during the 2^nd^ day following SD (Supplemental Figs S8 and S9).

To further investigate the extent of SD-induced alterations of neuronal activity in relation to sleep/wake states, we analyzed the dynamic of neuronal activity at different vigilance state transitions (Fig. 3). Although the activity in all structures maintained a vigilance state-dependent modulation with highest activity during REM sleep and waking, there was a general decrease in neuronal activity in all states after SD except for MUA during the transition wake to NREM sleep in the VTA that showed a non-significant increase during the 2th day after SD (Fig. 3). Collectively, our results show that SD induces long-lasting depression of the overall MUA in the VTA and SN which was evident in all vigilance states.

### Cortical EEG power densities are unreliable markers of MUA alterations in the VTA and SN

During NREM sleep, cortical activity is dominated by slow waves whose amplitude and incidence is quantified using spectral analysis of the EEG power density of cortical slow (0.1–1 Hz) and delta wave (1–4 Hz) oscillations^34^. This measurement -called SWA- is considered the most reliable index of sleep homeostasis^34,43^ and has been shown to reflect dynamic changes in neuronal activity in the cortex^35,44,45^. This also applies to the striatum under basal LD conditions^46,47^. Whether SWA dynamics correlate also with the alterations in neuronal activity that we found in the VTA and SN after SD is currently unknown. We therefore examined whether the changes in SWA faithfully mirror the SD-induced changes in neuronal firing in the VTA and SN. During baseline, and consistent with previous studies^30,35,44^, both slow wave (0.1–1 Hz, R^2^ = 0.61, p < 0.0001, polynomial regression analysis, Fig. 4A) and delta wave (1–4 Hz, R^2^ = 0.7, p < 0.0001, polynomial regression analysis, Fig. 4B) activity decreased during the day (i.e. the sleep phase) and increased during the night (i.e. the active phase). A similar pattern was also observed for the spindle frequency (7–15 Hz) activity (R^2^ = 0.67, p < 0.0001, polynomial regression analysis, Fig. 4C). After SD, both slow oscillations and delta wave activities were significantly higher than baseline for 6 and 5 hours respectively (Fig. 4A,B) while spindle frequency activity remained higher than baseline for 15 hours after SD (Fig. 4C). During the 2^nd^ day after SD, spindle frequency activity returned to baseline values (Fig. 4A–C). These results show that the dynamics of SWA do not reflect the electrophysiological alterations measured in the VTA and SN. Alterations in the latter were found to persist long after SWA returned to baseline (compare Fig. 2 with Fig. 4).

The correlation analysis between the changes in the delta-wave (1–4 Hz) activity and MUA during NREM sleep was either not significant (in the case of the VTA) or weakly positive in the case of SN-M and SN-L during both baseline and after SD (Fig. 5). The correlations with Slow oscillations (0.1–1 Hz) activity were positive after SD in both SN-M and SN-L (Fig. 5C,E). Although not significant during baseline in the VTA, this correlation was much weaker during post-SD1 and not significant during the 2^nd^ day post-SD (Fig. 5A). These results reveal a regional difference in the sensitivity of midbrain DA structures to sleep deprivation with the VTA being insensitive to increased sleep pressure relative to SN.

As an alternative, other frequencies in the EEG may better represent the long-term changes in neuronal activity in the VTA and SN^36,37^. Theta (6–9 Hz) power density during wakefulness showed a progressive increase during SD (Fig. 4D). After SD, it remained higher compared to baseline during the first 4h after SD (Fig. 4D). The power density of low beta (12.5–25 Hz) oscillations during wakefulness showed also a significant increase during SD but rapidly decreased to baseline value 1h after SD (Fig. 4E). The pattern of both theta and beta power density after SD also does not reflect the changes in neuronal activity observed in the VTA and SN during wakefulness (compare Fig. 4D,E with Supplemental Fig. S8). The correlations between changes in theta activity and beta activity with changes in MUA during wake were positive for both the SN-M and SN-L (Fig. 6B,C,E,F). For the VTA, the correlation with theta activity during baseline was much weaker or not significant after SD (Fig. 6A) while the correlations with beta activity were negative before and after SD (Fig. 6D). These results confirm the regional difference in the sensitivity and responses of the VTA and SN to different brain states. Furthermore, our findings indicate that although EEG power density correlated with neuronal activity in midbrain VTA and SN structures, the changes in the EEG merely mirror the long-lasting changes of neuronal activity patterns induced by SD in midbrain dopaminergic structures.

## Discussion

Here, we investigated the relationship between changes in environmental light, circadian and homeostatic components of sleep/wake behavior on the one hand and neuronal activity in midbrain’s VTA and SN on the other. A modest daily and circadian modulation of MUA was found in the VTA while in the SN, no significant circadian oscillation was found. Additionally, changes in the vigilance states was associated with a significant modulation of MUA in both the VTA and SN. The main finding of this study consists of the long-term alterations of MUA in these structures following a 6 h episode of sleep deprivation. Importantly, these alterations outlasted the changes in the density of cortical EEG waves which recovered from SD-related effects maximum 18 h following SD. This study shows that previously reported SD-related alterations within the reward circuitry^29^ could result from alterations in neuronal activity of midbrain DA structures precipitated by SD.

### Daily modulation of neuronal activity in the VTA and SN

The environmental light-dark cycle exerts a powerful synchronizing effects on physiological and behavioral rhythms^48^. To investigate the influence of LD on the rhythm of MUA as well as the potential circadian modulation of neuronal activity in the VTA and SN, we recorded under both LD and DD conditions. We found no significant daily and circadian oscillations of MUA firing rates in both subdivisions of SN while in the VTA a significant daily and circadian modulation of MUA was found. However, the amplitude of the MUA rhythms was about 6-fold lower compared to the robust rhythms reported in the SCN^41,42^.

Our recordings consist of multi-unit activity from the population of neurons surrounding the tip of the electrode regardless of the neurochemical identity of neurons. In both the VTA and SN, DA (≈70%) and GABA (30%) neurons are intermingled^1^. Therefore, the neural activity rhythms we recorded in these areas should be regarded as an estimation of the average activity of these two populations of neurons. A minority (2–3%) of neurons in VTA are glutamatergic^1^ and therefore, their contribution will be small compared to DA and GABA neuronal activity^49^.

Several monoaminergic neurons in the brain (e.g. norepinephrine, serotonin, acetylcholine and histamine) show robust vigilance state-dependent variation in firing rates, with high activity during waking and with pauses either in NREM or REM sleep^50^. In contrast, DA neurons in the VTA and SN do no significantly alter their firing rate across quiet wakefulness and sleep states^51–56^. Furthermore, within any vigilance state, DA neurons do not display a circadian modulation of their firing rates^51^. The only slight but significant change in firing rates of DA neurons in both the VTA and SN is a 20% increase in firing rates with active waking compared to all other states^51,52,55,56^. Vigilance state changes in firing patterns (regular vs bursting pattern) have been shown in the VTA^57^ and SN^58^. These alterations have been associated either with rewarding states^59^ or spontaneous movement^51,60^ during wakefulness. In contrast, GABAergic neurons undergo robust firing rate changes with sleep-wake vigilance states (50% and 130% increase in respectively active wake and REM sleep relative to SWS) in both the VTA^54,61^ and SN^54^. This specific vigilance state-dependent modulation of GABA neurons could also explain the low amplitude of MUA rhythms we found in both the VTA and SN given that GABA neurons account for only 30% of the total number of neurons in these structures. However, other factors such as weak neuronal connectivity with the central clock in the SCN could also account for the low amplitude of MUA rhythms. The absence of vigilance state-dependent variation in firing rate of DA neurons^51–56^ suggest that the robust rhythmic modulation of DA concentration in the brain is driven by other mechanisms such as the circadian modulation of DA re-uptake by DA transporters^62^.

We show that MUA rates in both VTA and SN undergo robust arousal-state-dependent changes with higher activity in REM sleep and wake compared to NREM sleep. This mirrors the pattern of changes in DA concentration in both the cortex and striatum^63^. The sharp changes seen in the NREM sleep to wake and wake to NREM sleep transitions are likely to reflect the sum of firing rate changes in presumed DA and GABAergic neurons^51,52,54–56,61^. The changes in MUA firing rates in SN at the NREM to REM sleep transition is likely to reflect changes in the activity of GABAergic neurons^54^ since DA neurons do not alter their firing rates during this transition^51–56^. In the VTA, recent electrophysiological^57^ and photometry recording^4^ showed a significant increase in activity when the animals transit from NREM to REM sleep. The significant increase in MUA in VTA at this transition is therefore likely to reflect the sum of increased firing rates of both DA^4,57^ and GABA neurons^61^.

### Light’s influence on MUA of the VTA and SN

The 24-h light-dark cycle has profound effects on multiple physiological functions^48^. Several lines of evidence support also a modulatory effect of the DA neurotransmission by light. For example, electrophysiological studies in rodents have shown acute increases or decreases of DA neuronal activity to brief light pulses^64,65^. Here we show that the 24 h dynamic of VTA and SN-MUA activity was not altered when the animals were released into one day of constant darkness. Furthermore, the dynamics of MUA at the vigilance states transitions were not significantly different between LD and DD. These results suggest that one day of DD has only minor effects on the electrophysiology of midbrain VTA and SN structures in mice. Long-term continuous exposure to bright light^66^ or constant darkness^67^ was shown to induce profound alterations of the DA neurotransmission including degeneration of DA neurons. These alterations negatively impacted mood by precipitating a depressive behavioral phenotype^67^. Additionally, long term exposure to different photoperiods affect different aspects of the DA neurotransmission in both rodents^68^ and humans^69,70^. Together with our results, these studies suggest that prolonged exposure to altered light schedules is necessary to adversely affect DA circuitry and its related physiological functions.

### Long-lasting effects of SD on neural activity of the VTA and SN

To investigate the electrophysiological responses of midbrain VTA and SN to increased sleep pressure, we sleep deprived mice for the first 6 h of the day during which mice normally spend most of their time asleep. Previous studies on the effect of sleep deprivation on DA neurotransmission have mainly used the stressful disk-over-water method to disrupt sleep^71,72^. This method makes it difficult to dissociate the observed alterations from the related effects such as stress associated with the method of SD^73^. Here, we used a method to stimulate spontaneous exploratory wakefulness by introducing fresh food and water and new objects into the cage without inducing stress^32,74^. The choice to perform the SD during the first 6 h of the subjective day is based on the rational of the study to investigate the impact of homeostatic sleep pressure on the activity of midbrain DA structures. Sleep pressure is the highest during this time window, hence a deprivation during this interval will maximally interfere with sleep need. Recent PET and fMRI studies in humans conducted in stress-free laboratory settings have reported a downregulation of dopamine D2/D3 receptors in the striatum following 24 h total SD^14,75–77^. These studies suggest that the DA neurotransmission is affected by SD per se^29^. By directly recording neuronal MUA in the VTA and SN, we extend these findings by showing that even a 6-h SD is sufficient to induce a significant and long-lasting decrease in neural activity in both the VTA and SN.

Because of our MUA neuronal recording method, we cannot pinpoint the neurochemical identity of neurons (DA or GABA) that reduced their firing rate after SD. One study employing single-unit recordings of DA neurons both in the VTA and SN in rats found no significant effect of SD on the firing rate of DA neurons^54^. In contrast, GABAergic neurons in the VTA showed a robust 40% decrease in firing rates following 24 h SD^61^. Thus, the reduced neuronal activity we found after SD in the VTA and SN could be attributed to reduced activity of GABA rather than DA neurons. This conclusion is corroborated by electrophysiological studies showing that GABA^61^ but not DA neurons^51–56,58^ display robust vigilance state-dependent modulations of their neuronal firing rates. Furthermore, recent microdialysis experiments in rats^77^ as well as PET studies in humans^14,76,77^ have shown that SD does not change DA content of the striatum which is one of the main target structures of DA neurons in both the VTA and SN. However, another study has reported increased extracellular DA in basal forebrain during, and throughout the 3 recorded hours following 6 h of SD^74^. Because basal forebrain is innervated also by a group of dopaminergic neurons located in the ventral periaqueductal gray matter (vPAG) which have been shown recently to modulate sleep-wake states^78^, the increase seen in extracellular concentration of DA in basal forebrain^74^ could be attributed to the activation of vPAG DA neurons^78^ rather than DA neurons in the VTA and SN^77,79^. Future studies using single cell recordings or *in-vivo* calcium imaging are needed to assess the responses of the different populations of DA neurons to SD as well as the relative contribution of DA vs GABAergic neurons to the altered electrophysiological activity we show here in the VTA and SN following SD.

### Cortical EEG and VTA and SN electrophysiology

Consistent with previous studies^34,44,80^, the dynamic of cortical waves as reflected by EEG power densities was altered by SD. We found that SWA, which comprises the EEG power density of cortical slow and delta wave oscillations in the range of 0.1–1 and 1–4 Hz respectively, increased during the 6 h following SD. Similar dynamic was also observed for theta wave activity during wakefulness while a more rapid recovery was observed for the beta wave activity during wakefulness. The increase in spindle frequency (7–15 Hz) activity lasted longer (up to 16 h after SD) (Fig. 4C). However, during the second day following SD, the dynamic of all EEG power densities recovered their baseline pattern. Given that the MUA changes in VTA and SN outlasted all EEG power density changes, the question arises why the altered neuronal activity precipitated by SD in the VTA and SN is not exteriorized at the EEG cortical level. Pharmacological^81–83^, transgenic^8^, as well as recent optogenetic^4,5,9^ studies have established conclusively the potent role of DA in the modulation of the electro-cortical activity over the different vigilance states. Therefore, the normal pattern of EEG signals (Fig. 4) as well as sleep/wake structure in the second day following SD would suggest an unaltered mesocorticolimbic DA neurotransmission. Microdialysis measurements of DA concentrations in rats^77^ as well as PET studies in humans^14,76,77^ support this conclusion. If the suppression of VTA and SN MUA reflects a decrease in the activity of GABAergic neurons in these two midbrain structures^61^, why is this not reflected at the EEG cortical level? To date, the physiological role of GABA neurons in midbrain VTA and SN structures in controlling particular EEG frequency bands has not been investigated^84^. Anatomical studies showed that GABAergic neurons in the VTA and SN project primarily to widespread subcortical areas^85^. The projections of VTA GABAergic neurons to the prefrontal cortex are sparse^85^. Therefore, the potential alteration of cortical neuronal activity as a result of SD-related suppression of VTA and SN-GABAergic neurons^61^ is unlikely to significantly alter EEG dynamic. Because of the dense subcortical projection of midbrain GABAergic neurons^85^, our results however, cannot exclude the possible alteration of neuronal activity (hence related-physiology and function) of potentially all subcortical targets of midbrain GABAergic neurons. For instance, GABAergic neurons in SN project densely to the thalamus^86^ and SD has been shown to significantly increase thalamic activity during the day following one night of total SD^26,27^. This hyperactivity response might reflect a disinhibition as a result of a suppression of GABAergic neurons in the SN after SD. Furthermore, we have recently demonstrated that 6 h SD induces a sustained suppression of MUA in lateral hypothalamus^80^ which receives also dense projections from VTA GABAergic neurons^85^ suggesting a causal link between these two structures in mediating SD-induced alterations of neuronal activity. Notably, our data also show that SWA, considered the most accurate index of sleep homeostatic pressure^34^ fails to reliably reflect the duration of changes in midbrain VTA and SN neuronal activity following SD. Taken together, our results show that SD induces long-term alterations of neuronal activity in the midbrain dopamine-GABAergic structures and that these alterations do not necessarily exteriorize at the cortical EEG level. Because we didn’t expect SD to induce such long lasting effects on VTA and SN electrophysiology, we recorded only 42 h following SD. Longer recordings will be necessary to study the complete dynamics of recovery. Such data holds also relevant translational value as to understand the long-lasting impact of SD on physiological functions related to midbrain DA and GABA systems.

### Potential mechanisms underlying the suppression of MUA in the VTA and SN

According to the activity-dependent metabolites homeostatic theory of brain function^50^, the increased metabolic rate during wakefulness is accompanied by the accumulation of specific metabolites in the extracellular milieu of the brain. These excess levels of metabolites (i.e. adenosine, GABA) passively act on the wake-promoting neuronal systems of the brain to dampen their activities (reviewed in^50^). Electrophysiologically, this leads to the slowing-down of the EEG signals corresponding to the initiation of sleep at the behavioral level. Although this phenomenon is not global, and regional differences in the sensitivity of different brain areas to these activity-dependent metabolites have been shown^87,88^, it has been demonstrated to operate in key wake promoting areas such as basal forebrain and lateral hypothalamus (reviewed in^50^). Here we show that the correlations between changes in MUA and delta wave activity after SD were either not significant (in the VTA and SN-M) or weakly negative (in SN-L). Furthermore, the correlations between changes in MUA during NREM sleep and slow oscillations activity after SD were paradoxically positive. The deeper NREM sleep was accompanied by higher MUA. Collectively these findings suggest that increased sleep pressure after SD contributes only weakly to the suppression of MUA we found in the VTA and SN following SD. This conclusion is also re-enforced by the persistence of the MUA suppression even after the recovery of both sleep/wake structure and the cortical EEG dynamic. Both DA and GABAergic neurons in the VTA and SN receive excitatory, inhibitory and modulatory inputs from diverse cortical and subcortical areas^89,90^. The activity of most of these structures is known to be affected by SD^27,28,88^. Therefore, the most parsimonious explanation of the electrophysiological alterations we found in the VTA and SN would be a local perturbation of the excitation and inhibitory balance because of SD-induced alteration of the activity of cortical and subcortical areas projecting to VTA and SN. Alternatively, SD is known to alter the expression of several neurotransmitter receptors which might affect neuronal excitability^91^. Whether this also applies to DA and/or GABAergic neurons in the VTA and SN is however not yet known.

### Clinical implications

DA and GABAergic neurons are part of a brain reward network^84,92^. The specific role of midbrain DA as well as GABAergic neurons in mediating reward-driven actions is well established^84,93^. Dysfunction of this system can lead to deleterious and life-threatening behaviors and emotional imbalance as exemplified by drug abuse and mood disorders^92^. In this study, we show that sleep states as well as sleep deprivation induce significant alterations of neuronal activity in midbrain VTA and SN. Perhaps the clearest implication of our results is the well-known association between sleep deprivation and aberrant reward-related behavioral outcomes^16–21^. Using fMRI, several studies have shown that SD leads to altered responses in several brain areas including the striatum and the VTA^17–20^. Importantly, these responses were associated with significant value computation biases as shown by greater emphasis on gain- relative to loss-related choices^17–21^. Furthermore, Volkow *et al*. have shown that SD induces a downregulation of striatal D2/D3 receptors in healthy volunteers^14,76,77^ which was associated with altered activation of a network of brain areas involved in attention^75^. Interestingly, the same downregulation of striatal D2/D3 receptors is also documented in cocaine abusers and short sleep duration has been recently suggested to significantly account for the relationship between cocaine abuse and the alteration of the striatal DA neurotransmission^94^. Our findings imply that midbrain dopaminergic structures are a potential neuronal center through which SD mediates these behavioral outcomes. SD has been also reported to confer a beneficial effect on motor impairments in PD patients^25^. However, this finding is not consistent and high degree of response-heterogeneity to SD was reported among PD patients^25^. Another clinical implication of our finding is the well-established link between SD and depression^34,95^. SD is a potent anti-depressive therapy in patients with depression^95^ and recently, optogenetic manipulations revealed a crucial role of VTA DAergic neurons in the rapid regulation of depression-related behaviors in mice^96^. Taken together, our results suggest that alterations of the activity of midbrain DA structures (VTA and SN) contribute significantly to changes in mood and motor functions and to reported deficits in judgement and decision making following sleep loss.

## Electronic supplementary material

Supplementary Information
