# Supplementary material for: Circadian and Homeostatic Modulation of Multi-Unit Activity in Midbrain Dopaminergic Structures
Source: Sci Rep. 2018 May 17;8:7765. doi: 10.1038/s41598-018-25770-5 (PMC5958140; doi:10.1038/s41598-018-25770-5)
Supplement: Supplementary file 1 — Supplementary Information [file 41598_2018_25770_MOESM1_ESM.docx]

**Circadian and Homeostatic Modulation of Multi-Unit Activity in Midbrain Dopaminergic Structures.**

Karim Fifel*, Johanna H Meijer & Tom Deboer

Department of Molecular Cell Biology, Neurophysiology unit, Leiden University Medical Center, P.O. Box 9600, 2300 RC Leiden, The Netherlands.

*To whom correspondence should be addressed:

Karim Fifel, Email: [fifel-k@hotmail.com](mailto:fifel-k@hotmail.com)

**Supplemental Figures**


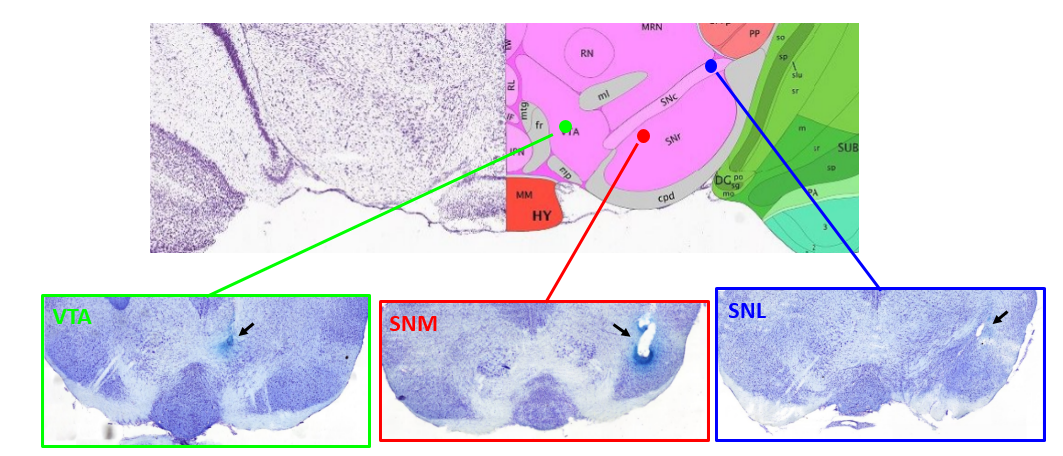


**Fig. S1. Depiction of examples of electrode placement in the ventral tegmental area (VTA), medial substantia nigra (SNM) and lateral substantia nigra (SNL) as confirmed by cresyl violet staining.** Atlas adapted from Allen Mouse Brain Atlas.


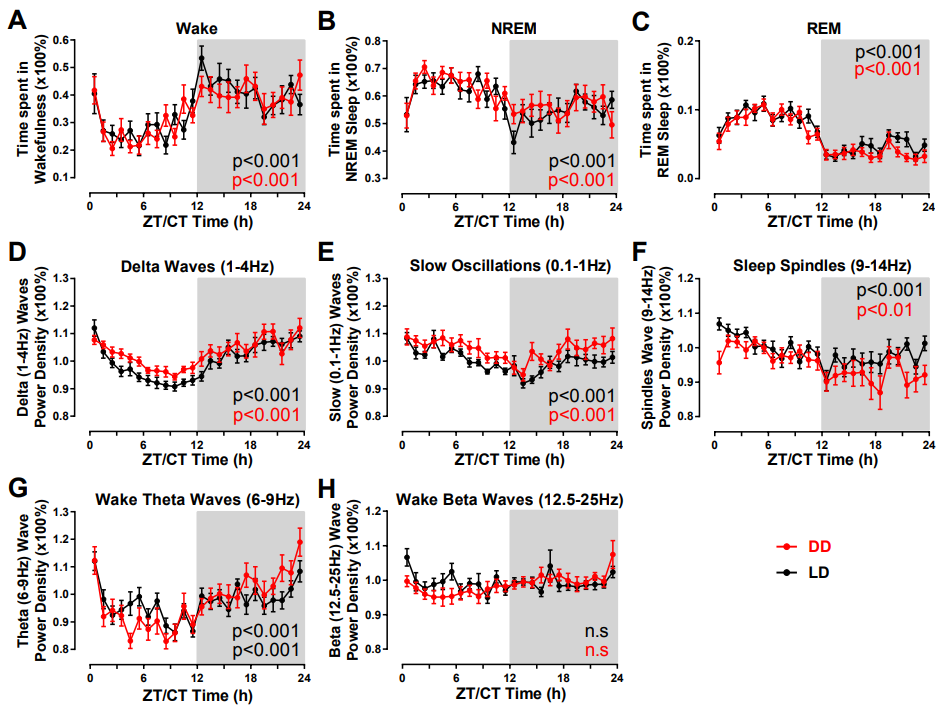


**Fig. S2. Evolution of sleep/wakefulness parameters and EEG power densities over LD and DD cycles.** (**A**) Wakefulness, (**B**) NREM sleep, (**C**) REM sleep, (**D**) Delta waves power density, (**E**) Slow oscillations power density, (**F**) Sleep spindles power density, (**G**) Theta activity and (**H**) Beta activity are presented as the mean ± s.e.m. Delta, Slow oscillations and sleep spindles power densities were extracted from EEG during NREM sleep while theta and beta power densities were extracted from EEG during wakefulness. The light (or subjective day in DD) and dark (or subjective night in DD) phases are indicated by white and black shading in the background. All values are expressed as a percentage of the mean activity measured during NREM sleep during LD cycle. All the rhythms are significant (One-way ANOVA and Cosinor analysis).


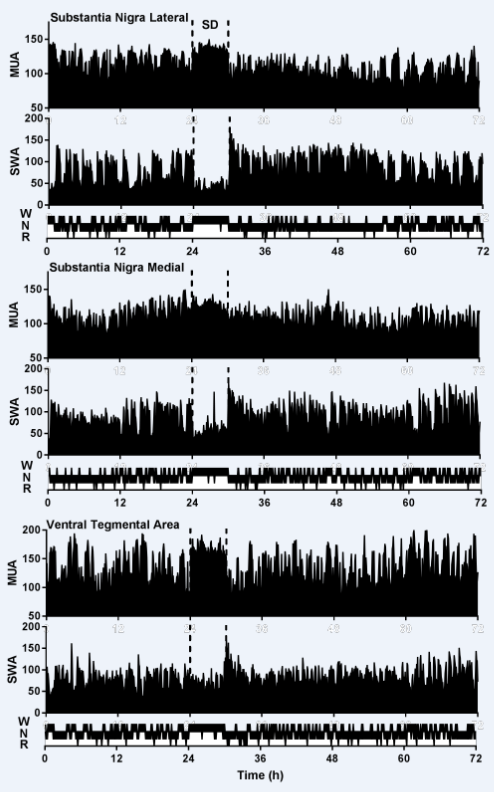


**Fig. S3.** Examples of individual traces of Multi-unit activity (MUA), slow wave activity (SWA) and vigilance states (W, Waking; N, non-REM sleep; R, REM-sleep) in lateral SN (Top 3 traces), medial SN (Middle traces) and the ventral tegmental area (bottom traces) over the three consecutive 24-hour periods of the experiment. Data for MUA, SWA and vigilance states are displayed in 5min intervals as a mean of thirty 10-s epochs. MUA and SWA are plotted as a percentage of the mean activity during NREM sleep over 24-h baseline day. Sleep deprivation (SD) was performed on the second day during the first 6 hours of the light period and is delineated by dashed lines in the graphs.


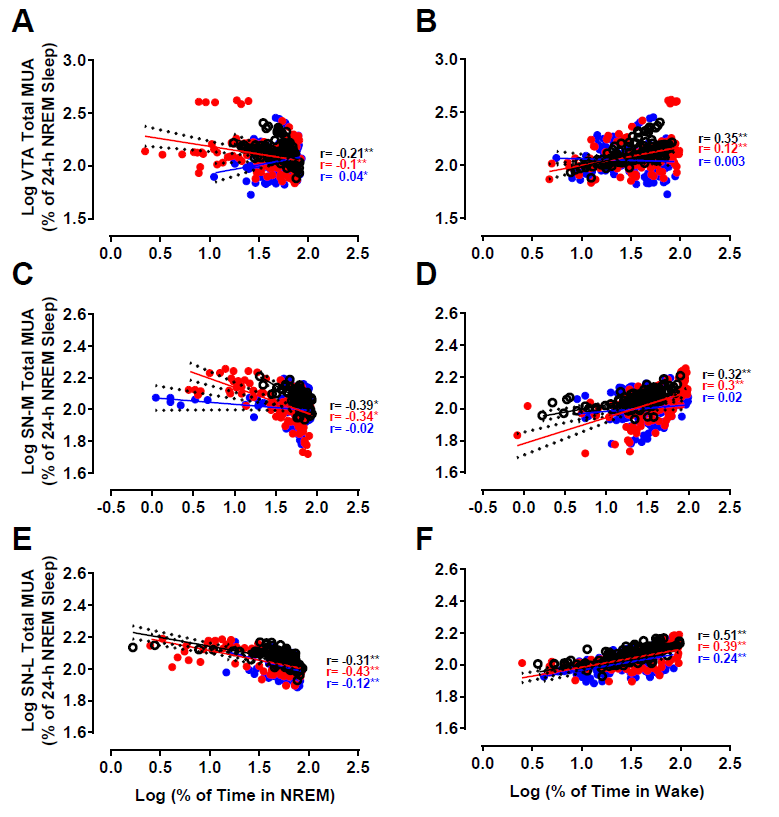


**Fig. S4. Correlation between neuronal activity in the VTA (A, B), SNM (C, D) and SNL (E, F) and vigilance states.** Mean firing rate in the VTA, SNM and SNL measured in 1-min bins as a function of the percentage of NREM sleep (**A**, **C**, **E**) and wakefulness (**B**, **D**, **F**). **p*<0.05, ****p*<0.001 (Black circles: Control day; Red circles: Post-SD1; Blue circles: Post-SD2).


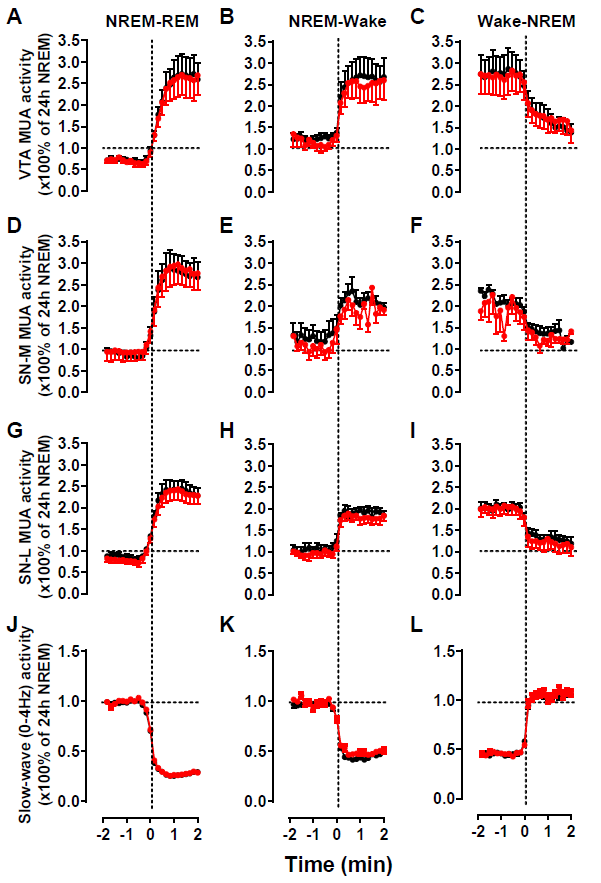


**Fig. S5. Multi-unit activity during vigilance-state transitions under both LD (black) and DD (red) conditions.** Time course of the VTA (**A**-**C**), SNM (**D**-**F**) and SNL (**G**-**I**) neuronal activity and EEG slow-wave activity (power density 0-4Hz) at the transition from NREM to REM sleep (**A**, **D**, **G**, **J**), NREM to REM sleep (**B**, **E**, **H**, **K**) and wake to NREM sleep (**C**, **F**, **I**, **L**) during the 2 min before and after the vigilance state transition. The curves connect 10s mean-values calculated over the entire LD and DD cycles. All variables are expressed as a percentage of the mean activity during NREM sleep over LD cycle. All changes at the transition were significant (*p*<0.001, ANOVA factor ‘time’ over 24 10-s epochs, Black lines: LD; Red lines: DD).


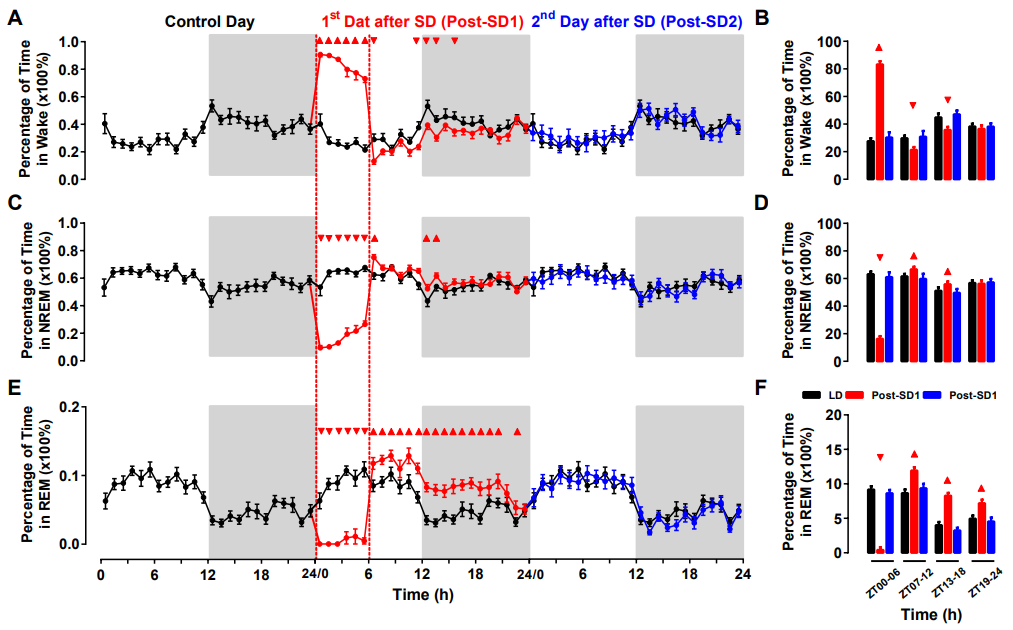


**Fig. S6. Changes in wake-sleep patterns following SD.** Wakefulness (**A**), NREM sleep (**C**), and REM sleep (**E**) were measured for 72 hours. The data are presented as the mean ± s.e.m. The light and dark phases of LD cycles are indicated respectively by white and gray shading in the background. The traces of the baseline day are triple-plotted in black for easy comparison. (**B**, **D**, **F**) Mean percentage of wakefulness (**B**), NREM sleep (**D**) and REM sleep (**F**) in 6h-bins. Triangles indicate significance with *p*<0.05 (two-way repeated measures ANOVA followed by Dunnett’s test).


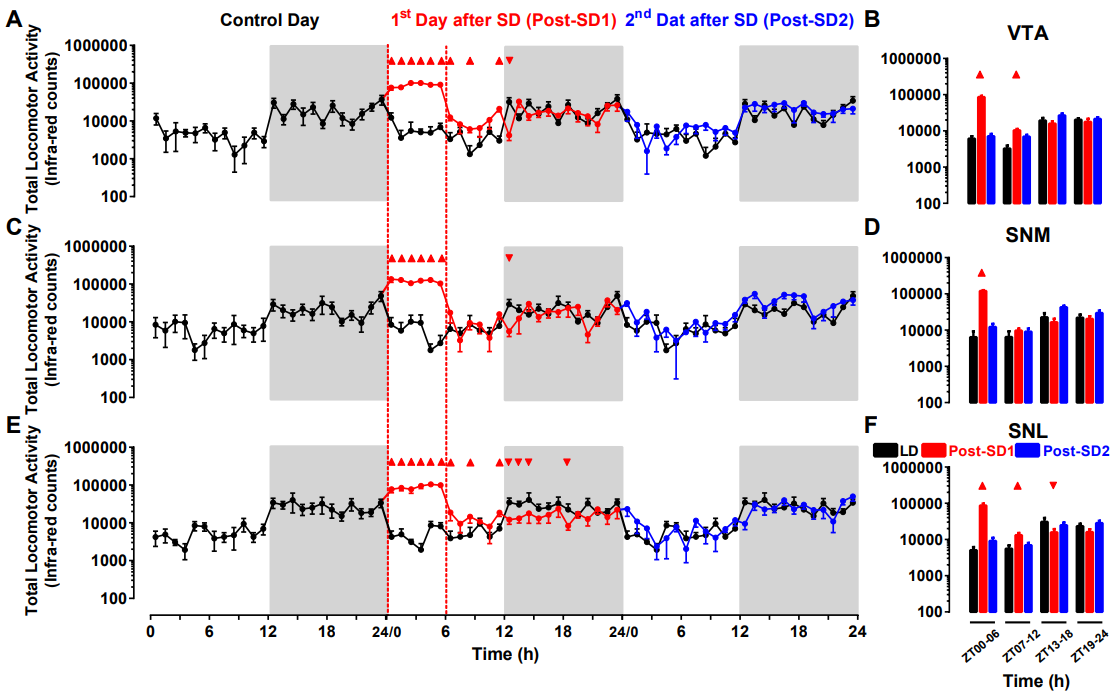


**Fig. S7. Changes in rest-activity rhythms over the 72 hours of the experiment in VTA (A, B), SNM (C, D) and SNL (E, F) animals.** The data are presented as the mean ± s.e.m. The light and dark phases of LD cycles are indicated respectively by white and gray shading in the background. The traces of the baseline day are triple-plotted in black for easy comparison. (**B**, **D**, **F**) Mean percentage of overall locomotor activity shwon in 6h-bins. Triangles indicate significance with *p*<0.05 (two-way repeated measures ANOVA followed by Dunnett’s test).


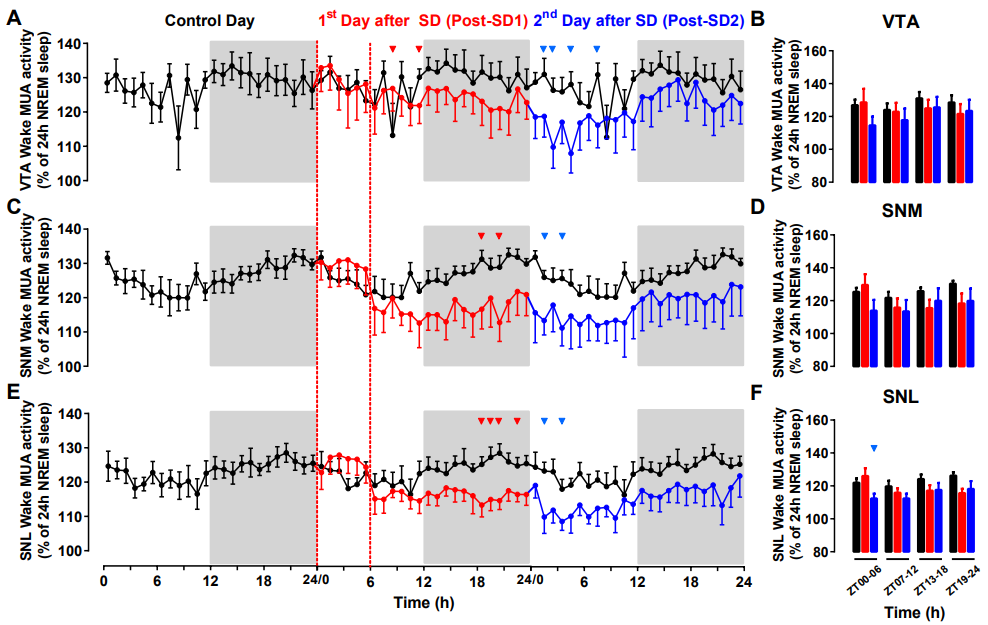


**Fig. S8. Changes in neuronal activity in the VTA and SN during wakefulness following SD.** Neuronal activity was measured during wakefulness in the VTA (**A**, n=6), SNM (**C**, n=5) and SNL (**E**, n=6) and are plotted as shown in Figure 2. (**A**, **C**, **E**) Activity is displayed in 1-hour intervals as a percentage of the mean activity measured during NREM sleep during the baseline day. SD was induced during the first 6 hours of the first day after baseline, and activity was measured during the 1^st^ (red) and 2^nd^ (blue) days after SD. For comparison, the data recorded during the baseline day in (**A**, **C**, **E**) are triple-plotted (black lines). The light and dark phases of LD cycles are indicated respectively by the white and gray shadings in the backgrounds. Error bars represent s.e.m. Triangles indicate significance with *p*<0.05 (two-way repeated measures ANOVA followed by Dunnett’s test). (**B**, **D**, **F**) Relative mean neuronal activity during wakefulness measured in 6h-bins in the VTA (**B**), SNM (**D**) and SNL (**F**) during control day (black bars), Post-SD1 (red bars) and Post-SD2 (blue bars). VTA, Ventral Tegmental Area; SNM, Medial Substantia Nigra; SNL, Lateral Substantia Nigra.


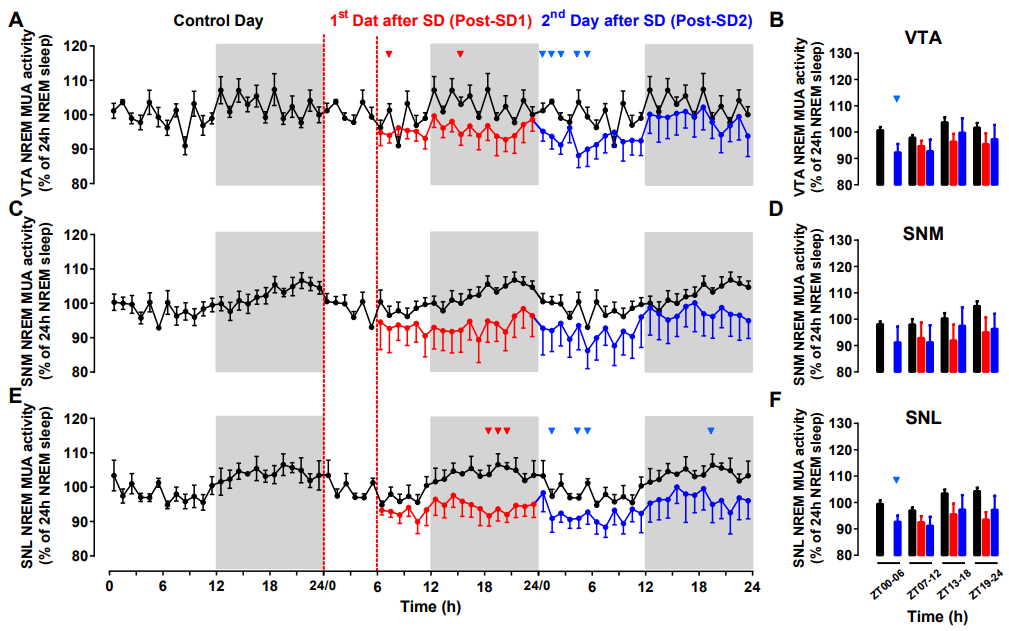


**Fig. S9. Changes in neuronal activity in the VTA and SN during NREM sleep following SD.** Neuronal activity was measured during NREM sleep in the VTA (**A**, n=6), SNM (**C**, n=5) and SNL (**E**, n=6) and are plotted as shown in Figure 2. (**A**, **C**, **E**) Activity is displayed in 1-hour intervals as a percentage of the mean activity measured during NREM sleep during the baseline day. SD was induced during the first 6 hours of the first day after baseline, and activity was measured during the 1^st^ (red) and 2^nd^ (blue) days after SD. For comparison, the data recorded during the baseline day in (**A**, **C**, **E**) are triple-plotted (black lines). The light and dark phases of LD cycles are indicated respectively by the white and gray shading in the background. Values during the 6h SD are omitted because the remaining ≈10% of NREM during this period are influenced by the SD intervention. Error bars represent s.e.m. Triangles indicate significance with *p*<0.05 (two-way repeated measures ANOVA followed by Dunnett’s test). (**B**, **D**, **F**) Relative mean neuronal activity during NREM sleep measured in 6h-bins in the VTA (**B**), SNM (**D**) and SNL (**F**) during control day (black bars), Post-SD1 (red bars) and Post-SD2 (blue bars). Values during the 6h SD are omitted because the remaining ≈10% of NREM during this period are influenced by the SD intervention. VTA, Ventral Tegmental Area; SNM, Medial Substantia Nigra; SNL, Lateral Substantia Nigra.
